# Natural Killer Cells in the Malignant Niche of Multiple Myeloma

**DOI:** 10.3389/fimmu.2021.816499

**Published:** 2022-01-11

**Authors:** Ondrej Venglar, Julio Rodriguez Bago, Benjamin Motais, Roman Hajek, Tomas Jelinek

**Affiliations:** ^1^ Faculty of Science, University of Ostrava, Ostrava, Czechia; ^2^ Faculty of Medicine, University of Ostrava, Ostrava, Czechia; ^3^ Hematooncology Clinic, University Hospital Ostrava, Ostrava, Czechia

**Keywords:** NK cells, multiple myeloma, inhibitory receptors, activating receptors, immunotherapy, microenvironment, niche

## Abstract

Natural killer (NK) cells represent a subset of CD3- CD7+ CD56+/dim lymphocytes with cytotoxic and suppressor activity against virus-infected cells and cancer cells. The overall potential of NK cells has brought them to the spotlight of targeted immunotherapy in solid and hematological malignancies, including multiple myeloma (MM). Nonetheless, NK cells are subjected to a variety of cancer defense mechanisms, leading to impaired maturation, chemotaxis, target recognition, and killing. This review aims to summarize the available and most current knowledge about cancer-related impairment of NK cell function occurring in MM.

## Introduction

Multiple myeloma (MM) is a malignant disorder of plasma cells (PCs) with a median age of 65 years at diagnosis. MM evolves from monoclonal gammopathy of undetermined significance (MGUS) present in >3% of the population aged >50 years (1). The disease’s clinical manifestations are mostly elevated serum calcium, renal failure, anemia, and bone involvement (the acronym CRAB) (2). Eventually, in up to 20% of cases, MM can progress into extramedullary disease (EMD), a soft tissue plasmacytoma, which represents a highly aggressive and treatment-resistant stage of MM (3–6). The mechanisms and biology of EMD are poorly understood, though PCs accumulate more chromosomal aberrations during EMD transformation (7).

Due to their natural tumor suppressor potential, natural killer (NK) cells became a subject of intensive research in cancer immunotherapy in both solid tumors and hematological malignancies (8, 9). Restoring or enhancing the effector abilities of NK cells for the treatment of MM has been one of the key topics in recent years (10–12). NK cell therapy is advantageous for several reasons: (1) NK cells are easy to isolate and expand *in vitro* using well-established methodologies; (2) these cells are capable of both direct killing and secretion of cytokines that can either potentiate other immune cells or suppress tumor cells; (3) overall biological features of NK cells are reducing the possibility of undesired side effects such as the ones observed with CAR-T cells; (4) NK cells are not antigen specific and there is no need for a specific target, although this can enhance the effectiveness of the therapy (13–15); and (5) the infusion of allogeneic NK cells is safe and does not cause the unwanted and deleterious graft vs. host disease (GvHD), thus opening the possibility of a more affordable off-the-shelf cancer cell-based immunotherapy (16).

Novel NK cell-based therapy possibilities include infusion of allogeneic/autologous NK cells, administration of *in vitro* expanded and genetically modified NK cells (including CAR-NK cells), cytokine-stimulated NK therapy, and monoclonal antibody (mAb)-based NK therapy (13, 15, 17). Modification of inhibitory or activating surface molecules represent a promising option to potentiate efficacy of NK cells (18, 19). Another promising approach is priming of NK cells with certain interleukins (ILs). IL-2 and IL-15 supplementation *in vitro* was confirmed to enhance the NK cells’ killing abilities, increasing the expression of activating NK cell receptors (20–22). Although the NK therapy seems to hold a huge potential for cancer therapy, a recent study showed that haploidentical NK cell transplantation in relapsed/refractory (RR) MM patients did not report significant therapeutic outcomes. The study had to be halted after all 12 patients relapsed within 90 days (23). Also, it is important to understand that mAb therapies for the treatment of MM act through (amongst others) NK-cell activities like antibody-dependent cell cytotoxicity (ADCC) mediated *via* either the mAb or mAb–drug conjugate (24–26). Anti-CD38 daratumumab (approved in 2015), anti-SLAMF7 elotuzumab (2015), and anti-CD38 isatuximab (2020) are mAbs used for the treatment of MM (25, 27, 28). The novel anti-CD38 MOR202 is now in the clinical trial phase in MM patients (29). Likewise, proteasome inhibitors and immunomodulating agents such as thalidomide, lenalidomide, and bortezomib have been proved to potentiate NK cell activity against MM (28).

Understanding the NK cell biology and mechanisms affecting the function of NK cells in MM is crucial for further progress in the field of targeted and NK cell therapy. This review summarizes the most recent and available data providing a necessary insight into the origin and development of NK cell subsets, their biology, antitumor abilities, and, mainly, impairment of function occurring in the MM microenvironment.

## NK Cell Development and Subsets

**Figure 1:**
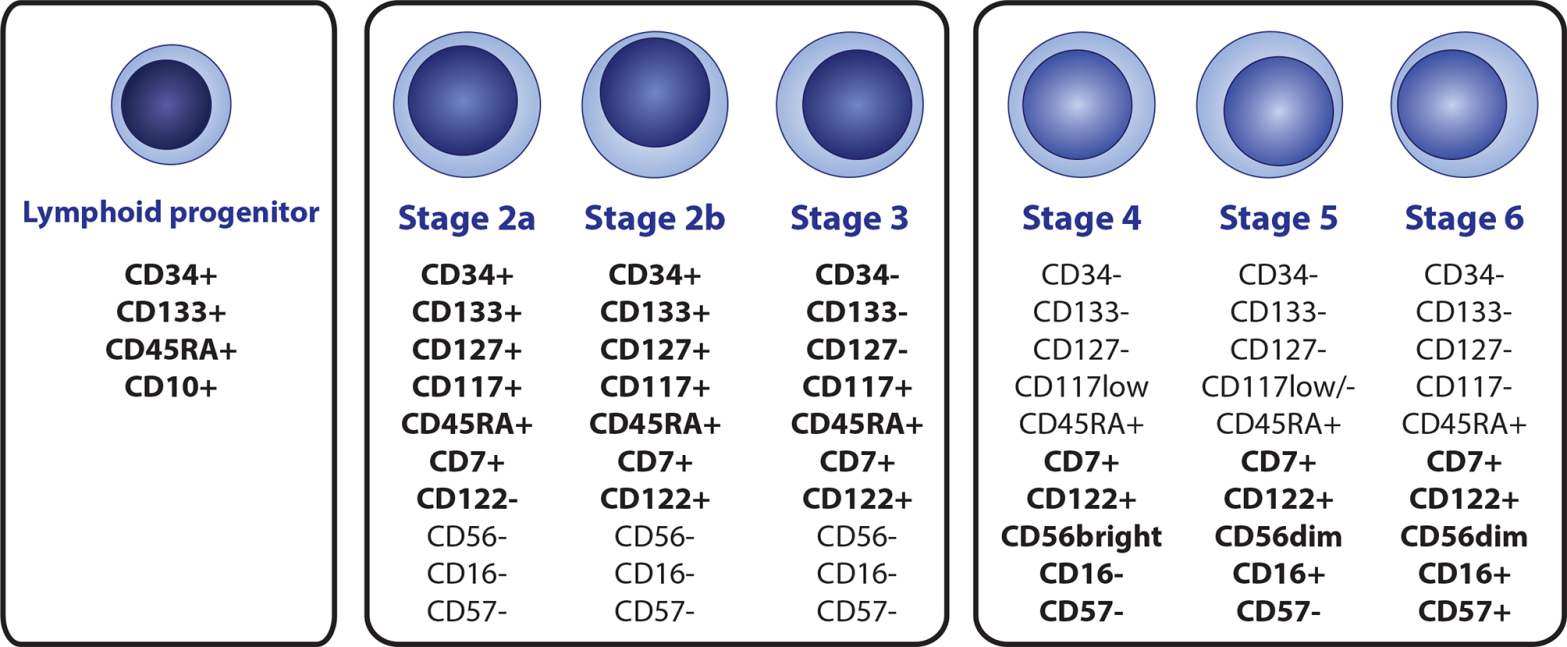
A scheme of the NK cell development, with immunophenotypic profile of the most relevant markers that are suitable for identification of individual subsets by flow cytometry. NK cell lineage is derived from the CD34+ CD133+CD45RTA+CD10+ lymphoid progenitor (terned also as a stage 1), Immature stages 2a, 2b and 3 can be distinguished by the differential expression of CD34, CD122 and CD127, while high levels of CD117 are preserved. Mature stages 4 (4a + 4b), 5, and 6 can be distinguished by diverse expression of CD16, CD56 and CD57.

NK cells represent 2–31% of peripheral blood (PB) lymphocytes (30). Although the organ and tissue distribution and circulation of NK cells are not fully understood, they are also present in the bone marrow (BM), liver, spleen, lungs, uterus, thymus, and secondary lymphoid tissues (31, 32). Maturation and differentiation of early NK subsets occurs in BM and secondary lymphoid organs. Even though NK cell development in humans is understood less than in mice, stages 1 to 6 were identified (8 overall with substages) in humans, each having a distinct immunophenotypic profile (**Figure 1**) (33, 34). Several ILs are crucial for the development of the NK lineage, mainly IL-2, IL-7, and IL-15, but also pro-inflammatory IL-12, IL-18, IL-27, and IL-35 (35, 36).

Similar to all other hematopoietic lineages, the NK lineage is derived from bone marrow-residing hematopoietic stem cells (HSCs), which transition into CD34+ CD45RA- CD133+ multipotent progenitor (MPP) cells and subsequently to CD34+ CD133+ CD45RA+ lymphoid-primed multipotent progenitors (LMPPs), determining the lymphoid line potential. Direct NK cell lineage precursors seem to be derived from the CD34+ CD133+ CD45RA+ CD10+ fraction, known as common lymphoid progenitors (CLPs) in the conservative model of hematopoiesis, or multi-lymphoid progenitors (MLPs) according to the proposed hematopoietic tree revision. The revision does not distinguish NK lineage potential and further NK cell development since the study aimed at early CD34+ progenitors and also mainly on the revision of the general myeloid and lymphoid progenitor potential (35, 37, 38). The earliest NK precursors with acknowledged NK lineage potential were identified as CD127+ in mice (39). This correlates with CD34+ CD133+ CD45RA+ CD7+ CD117+ CD127+ stage 2a phenotype in humans. CD7 (from the stage 2a) and CD122 (from the stage 2b) are subsequently expressed throughout the whole NK cell lineage. Stage 3 represents a transitional stage between NK precursors and mature NK cells with a complete loss of CD34, CD133, and CD127 but with prevailed high levels of CD117  (35, 40–42). Mature subtypes of NK cells (stages 4a, 4b, 5 and 6), all with the characteristic CD3- CD7+ CD45RA+ CD56+/dim immunophenotype are characterized by the progressive loss of CD117 from high to low levels in stage 4 to stage 6 (the final stage) being completely CD117-, and with the gain of CD56 (33, 43, 44).

CD56 and CD16 represent two of the most common and relevant markers used to identify NK cells (32, 45). For a proper flow cytometric detection of all mature NK cell subsets, both CD56 and CD16 should be included in the panel since CD16 is expressed only in stages 5 and 6, and CD56 alone is not sufficiently specific (32). CD3 should also be mandatory for correct NK cell evaluation to exclude CD3+ CD7+ CD56+ NK-like T cells (46). Based on the expression of CD56 and CD16, two main mature functional subsets are often described: CD56+/bright CD16-/dim and CD56dim CD16+/bright (47). CD56+ CD16- cells (accounting for 5-10% of circulating NK cells) are agranular with low cytotoxic activity and are considered mainly to be cytokine and chemokine producers. These cells co-express CD94/NKG2A in a high manner, meaning the CD56+ CD16- subset consist of both stages 4a and 4b. Contrary to this, CD56dim CD16+ cells (90%–95% of circulating NK cells) are designated as true killer cells with a high cytolytic potential against infected, tumor-transformed, or otherwise immunocompromised cells due to the expression of CD16 (Fcγ receptor III), which acts as a cell lysis signal transducer. A typical feature is the diminished expression of CD94/NKG2A compared to high levels of this antigen on the surface of CD56+ CD16- cells (45, 48–50).

CD57+ is a terminal marker of CD8+ T cells and also NK cells (51). The CD56dim CD16+ subset consists of developmental stages 5 and 6. The main difference in these two stages is in the expression of CD57 and regulatory surface molecules known as the Killer cell Ig-like Receptors (KIRs); stage 5 lacks CD57 and maintain only low levels of KIRs (NK stage 5 immunophenotype: CD56dim CD16+ CD57- KIRdim), whereas the terminal stage 6 expresses both CD57 and KIRs in a high manner (NK stage 6 immunophenotype: CD56dim CD16+ CD57+ KIR+) (32, 35).

The CD56- CD16+ subset was also identified in high numbers in individuals with chronic infections (HIV and HCV). The subset is described as dysfunctional, with higher expression of NK inhibitory receptors, lower levels of NK cytotoxic molecules, and both limited cytotoxic function and secretion of anti-inflammatory cytokines (52, 53). NK subsets can also be described by different levels of CD27 and CD11b in both mice and humans (54, 55).

Although there are several known and accurately described subsets of NK cells, it seems that diversity in the expression of different NK surface molecules pushes the variability of NK cells beyond the limits of standard flow cytometry. Between 6,000 and 30,000 different NK phenotypes can be detected in one individual and up to 100,000 in a group of individuals using the mass cytometry approach (56).

## NK Cell Biology in Anticancer Immunity

The role of NK cells in anticancer surveillance is unquestionable in the modern era. Many studies have highlighted the significance of NK cells in the elimination of malignant cells or in cancer progression regulation, a topic that has been heavily reviewed in recent years (49, 57, 58). NK cells were originally categorized as part of the innate immunity; however, memory and education abilities have been a matter of discussion lately (59). These cells lack specific antigen receptors compared to other lymphocyte subsets. The anti-cancer potential of NK cells is mediated either directly in a contact-dependent manner through their ability to induce programmed cell death, or indirectly in a contact-independent manner through the secretion of various cytokines, or in both manners by cooperation with other cells of the immune system (60). A broad spectrum of surface regulatory molecules is involved in NK regulatory actions (61).

### Anticancer Mechanisms of NK Cells

The release of cytokines and chemokines, which are soluble, omnipresent, and crucial immune system regulators, is one of the key antitumor abilities of NK cells (48). The CD56+ CD16- NK cell subset is considered a major cytokine producer with low killing abilities (47). Nonetheless, CD56dim CD16+ cells, otherwise with a strong cytolytic potential and present in the majority in peripheral blood, also act as cytokine producers mainly in the initial immune response, which helps in mobilizing other immune cells (62). Tumor necrosis factor α (TNF-α) and interferon γ (IFN-γ) are among the most potent antitumor cytokines, but the NK cell cytokine repertoire also includes immunoregulatory IL-5, IL-10, and IL-13; chemokines CCL2 (MCP-1), CCL3 (MIP-1α), CCL4 (MIP-1β), and CCL5 (RANTES); and GM-CSF as well (63).

The ability to induce apoptosis of the target cell is a primary and well-known regulatory mechanism of NK cells. Apoptosis induced by NK cells can be mediated by degranulation, death receptors, or mAb-CD16 binding (60). The degranulation ability of NK cells was proved to be crucial in tumor and metastatic regulation (64). A specialized organelle called secretory lysosome, mainly containing perforin and granzyme granules, is involved in the highly coordinated and regulated process (65, 66). Death receptors are TNF superfamily receptors expressed on the surface of many cells (67). Death receptor ligands expressed by NK cells (such as Fas ligand, TNF, and TRAIL) bind specifically to the death receptor domains on the surface of target cells, resulting in a conformational change of the receptor, recruitment of the adaptor protein, and apoptosis (65, 68). ADCC represents one of the cancer immunotherapy-related killing mechanisms of NK cells. ADCC is facilitated after the binding of an IgG mAb Fab fragment to the target surface antigen on one side and Fc fragment to the Fcγ receptor III (CD16) of the effector cell on the other side, creating the effector cell–mAb–target cell link with subsequent engagement of cytotoxic pathways (69).

### Surface Effector Receptors

NK cell surface activating and inhibitory molecules play a crucial role in the regulation of NK cell killing abilities, cytokine production, and all actions, in general. These receptors are able to detect specific stress signals and changes in expression patterns of surface molecules on cells and consequently regulate NK cell activity, which is a deeply balanced process (61, 70). During differentiation, NK cells undergo a complex series of educational interactions between major histocompatibility complex type I (MHC-I) molecules and NK surface inhibitory receptors. Thus, they are educated to self-tolerate other healthy cells in the body (71, 72). Also, interactions between non-classical MHC and non-MHC molecules were described (73). The concept of induced tolerance and inhibition of NK cell activity by recognizing MHC is fundamental for the regulation of the anti-cancer response. During malignant transformation, a series of changes in gene expression occur in transforming cells, leading to the downregulation or upregulation in the expression of surface molecules (74). In this context, the most important is the diminution of surface MHC-I molecules, which tags transformed cells as a potential target for eliminating NK cell-regulatory mechanisms (75, 76).

**Figure 2:**
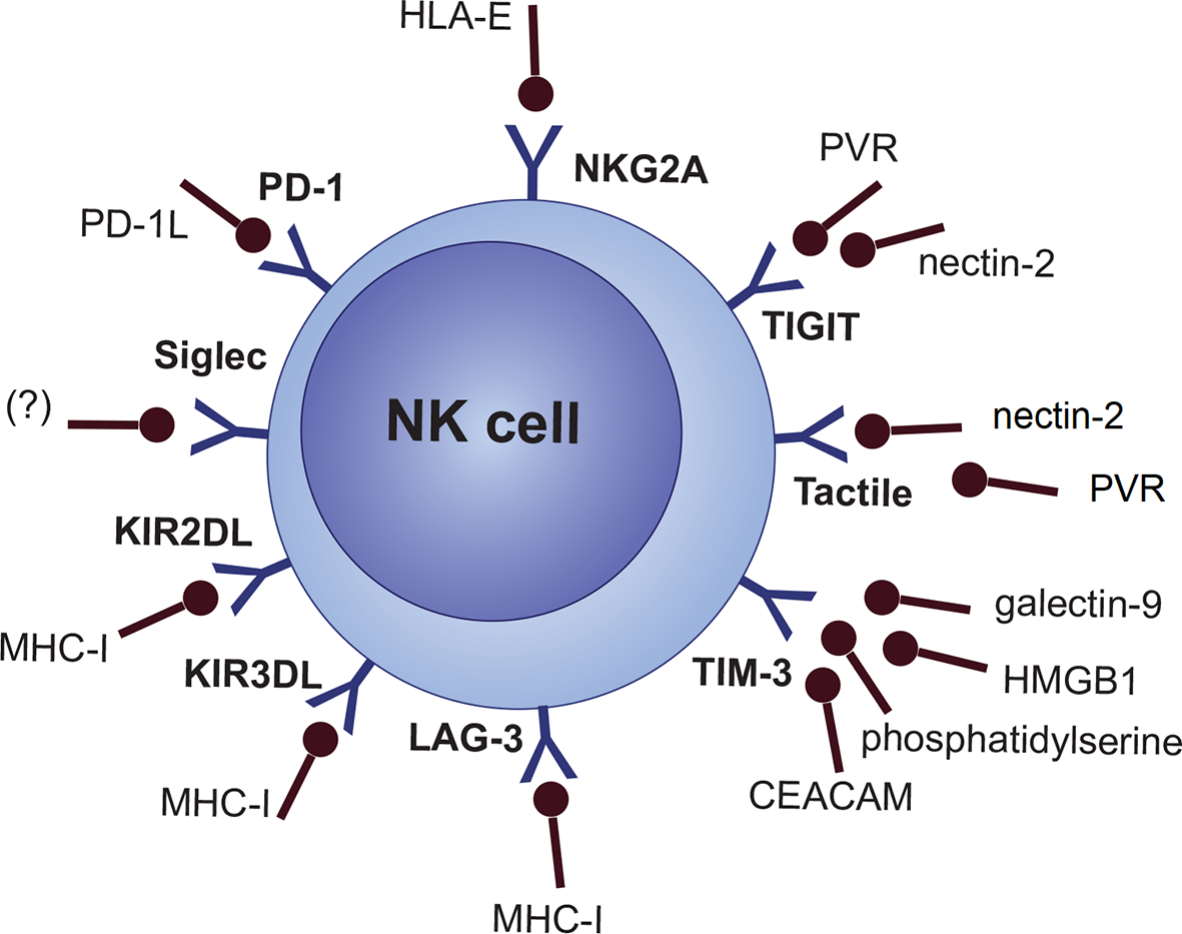
NK cell inhibitory receptors will their cognate ligands.

NKG2A/CD94 heterodimer (CD159a), LAG-3, and a fraction of the killer Ig-like receptor (KIR/CD158) family (inhibitory KIRs [KIR2DL, KIR3DL subgroups]) are categorized as specific MHC-I/HLA-I recognizing inhibitory NK cell receptors (61, 77, 78). However, this does not necessarily mean that any cells lacking MHC-I are the target of NK cells. There are several other inhibitory and co-inhibitory NK cell molecules like the Siglec family (e.g., Siglec 7 and Siglec 9), Tactile (CD96), PD-1, TIGIT, CD112R, IL-1R8 and TIM-3 (**Figure 2**) (77, 79, 80).

**Figure 3:**
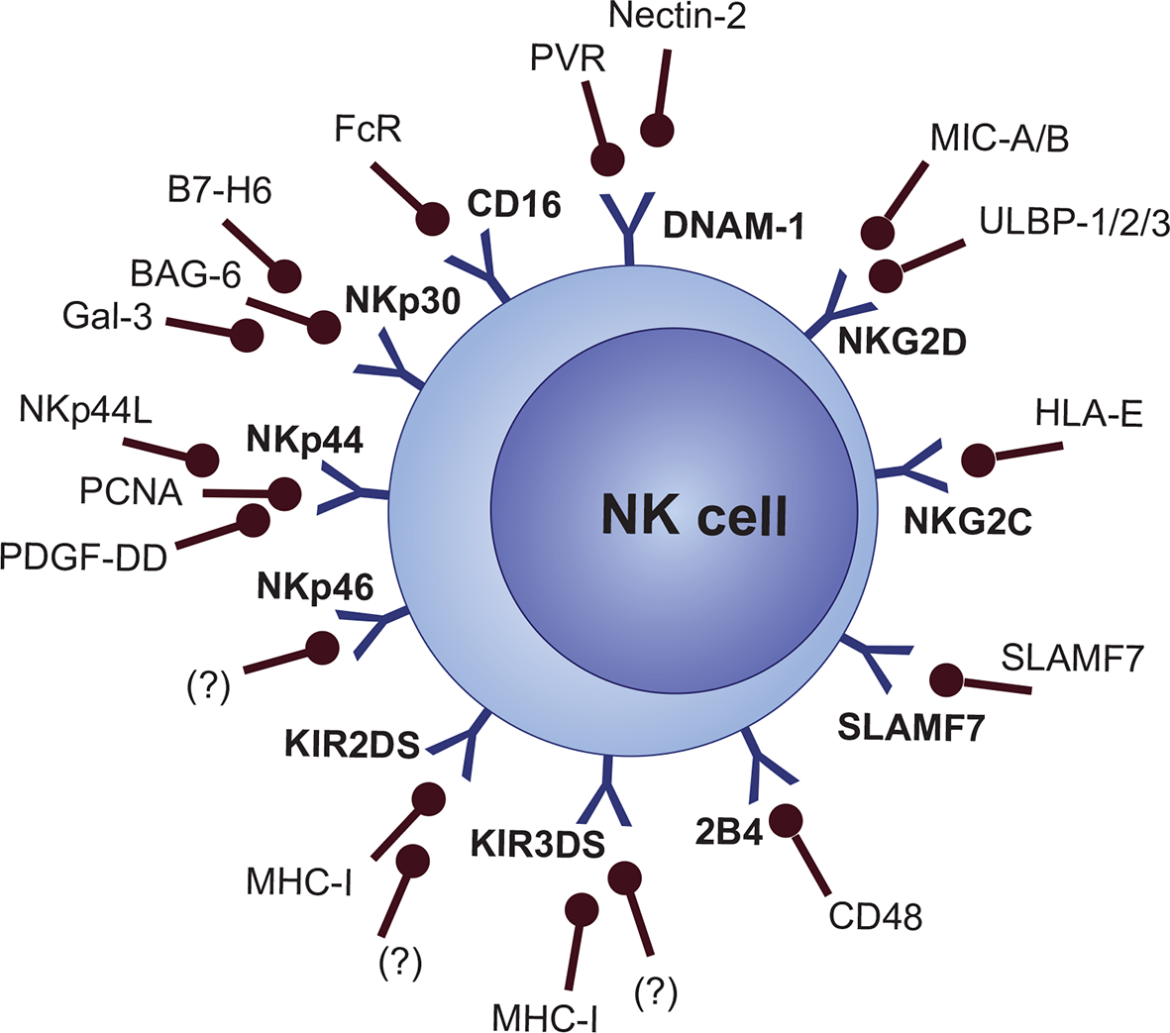
NK cell activating receptors and their ligands.

Nevertheless, a “missing-self” signal is not enough for the activation of NK cells. Expression of stress-induced signals which stimulate the NK cell-activating receptors is crucial for activating NK cell response (81). Cellular stress activates a variety of DNA-damage response, senescence, and tumor-suppressor signaling pathways, which consequently lead to the expression of activating ligands that are recognized by NK cell activating receptors (82). Also, the synergistic action of multiple activating molecules is required for the activation of NK cells, except for CD16 and NKG2C, which are able to activate cell response on their own without any other co-stimulation (19, 83, 84). Several MHC-dependent and MHC-independent molecules are categorized as NK cell activating receptors, including activating KIRs (KIR2DS and KIR3DS subgroups), NKG2D, NKG2C, natural cytotoxicity receptors (NCRs [NKp30, NKp44, and NKp46]), Nkp80 (not clearly categorized as NCR), ICOS, DNAM-1 (CD226), CRTAM, and signaling lymphocyte activation molecule (SLAM) family members like 2B4 (CD244), CD48, Ly9 (CD229), NTB-A (CD352), and SLAMF7 (CD319) (**Figure 3**) (19, 85–92).

Originally, functional receptors of NK cells were categorized either as inhibitory or activating, but there are hints that the function of some molecules might be much more complex with a dual inhibitory and activating potential or at least a costimulatory function (13). For example, both the inhibitory and activating potential of 2B4 (CD244) was proved (93). There seems to be evidence that the activating molecule NKG2D also has broad costimulatory abilities of other activating receptors (94).

Other molecules are of course present on the surface of NK cells, but they are not clearly categorized among the activating or inhibitory receptors. Nevertheless, CD38, which is an important signal transducing, activating, and adhesion molecule, was also proved to activate NK cell effector response (95, 96). CD27, a T-cell co-stimulatory molecule, is not mentioned similarly in this context, but CD27 was connected with the enhanced cytotoxic activity of NK cells (97).

## MM Microenvironment

The BM niche, in general, is a deeply complex environment, which consists of cellular and noncellular components. The cellular compartment is represented either by hematopoietic cells, or nonhematopoietic cells such as mesenchymal stromal cells (MSCs), osteolineage cells, adipocytes, and endothelial cells. Cytokines, chemokines, growth factors, reactive species, extracellular matrix (ECM) proteins, and other molecules form the noncellular compartment (98, 99). BM function is negatively affected in hematological malignancies due to the tumor microenvironment (TME), which creates advantageous conditions for clonal cells and suppressive conditions for normal cells. In MM, disease manifestation, progression, and treatment resistance are often reflected with TME and its individual components (100, 101). Single-cell transcriptomics data revealed that alteration in the immune setup of the BM niche can be observed early from the MGUS stage, including increased frequency of NK cells, T cells, and monocytes. T cells exhibit accumulation of Treg and γδ T-cell subsets at MGUS, accompanied by decrease of CD8+ memory subsets at the stage of SMM. Importantly, the patterns of immune dysregulation are heterogeneous in MM patients and might represent a possible indicator for the risk stratification (102).

### Non-Cellular Compartment

The role of cytokines, growth factors, extracellular vesicles, and other molecules was described in the process of TME transformation and MM progression (103). Furthermore, the presence of tumorigenic molecules plays a critical role in the concept of pre-metastatic niche describing slow and remote TME orchestration, connected to the disease dissemination (104, 105). Malignant BM is highly inflammatory and hostile to non-malignant cells (including NK cells), a fact that is reflected by elevated levels or altered expression of pro-inflammatory factors such as IL-1, IL-6, IL-10, IL-17, IL-18, IL-21, IFN-γ, TGF-β, TNF-α, HGF, EGF, and HIF-1α; chemotactic factors recruiting other pro-inflammatory cells such as CCL2, CCL3, CCL4, CXCL12 (SDF-1), CSF-1, GM-CSF, and MSP; and proangiogenic factors supporting neovascularization such as VEGF, IGF-1, FGF, PDGF, reactive oxygen species (ROS), and reactive nitrogen species (RNS). The overall profile of soluble factors is a deeply complex topic itself (106–110).

### Cellular Compartment

Normal cells present in the BM stroma can be clearly reprogrammed to support the disease manifestation and progression. This is reflected by the fact that immature BM MSCs have an abnormal genomic profile compared to their normal counterparts and provide advantageous environment for the expansion of MM cells (111, 112). Hence, the role of MSCs in disease persistence was suggested. Single-cell transcriptomic analysis revealed that MSCs in MM are highly pro-inflammatory, their transcriptomic profile can be tracked even post-treatment, and unfortunately, therapy is not effective in normalizing the BM niche. The study also revealed that MM MSCs are stimulated by pro-inflammatory cytokines that are most likely produced by immune cells such as IFN-responsive T cells and CD8+ memory T-cell subsets (113). MSCs development is also disrupted in MM, and aberrancies were described in more mature osteolineage cells and adipocytes (114, 115). The differentiation of MSCs is shifted preferentially towards the adipocyte lineage in MM, and, if the high secretory potential of adipocytes is taken into consideration, this may favor further disease progression as well (116). *EPHB1, FBLN5, RELL1, ADAMTS17* are among the impaired genes in MM-affected MSCs. Downregulation of *BMP10*, the bone morphogenic protein 10 gene, in MM MSCs reflects the impaired osteoblastic differentiation, and it seems that BMP signaling is involved in MM bone disease progression. Therefore, inhibition of the BMP axis, as well as others such as TGFβ, Notch, Wnt, or Runx2/Cbfa1 signaling, represents a possible option for therapy improvement in MM (112, 117, 118). Interestingly, interactions between MM cells and BM MSCs trigger the production of IL-6 and a number of cytokines and chemokines, including TNF-α, VEGF, IGF-1, CXCL12, IL-1β, TGF-β, CCL-3, and CCL4 with immunomodulatory activity (119, 120).

Over-angiogenic potential of endothelial cells (ECs) was linked with neovascularization and disease progression in MM (121). ECs of MM patients also have a distinctive genetic profile that strongly supports their neoangiogenic potential. Genes involved in neovascularization, such as *bFGF, FGF-7, VEGF-A, VEGF-B, VEGF-C, VEGF-D*, and *GROα*, together with ETS-1, HIF-1α, ID3, and osteospontin transcription factors, are overexpressed in MM ECs (122). Also, filamin A, vimentin, and α-crystallin B proteins are overexpressed by MM ECs, though anti-MM drugs such as bortezomib and lenalidomide affect these proteins during treatment (123). The hypoxic niche and HIF-1α overexpression are key factors in MM neovascularization as well (124).

The most relevant hematopoietic cells contributing to the MM TME are without a doubt malignant PCs, macrophages, myeloid-derived suppressor cells (MDSCs), and T-regulatory lymphocytes (Tregs) (105). Overall impact of malignant PCs can be seen throughout the whole chapter, but briefly, the role of PCs in the organism is much more complex than just antibody production. They are able to produce many soluble factors, including IL-1, IL-10, IL-12, IL-17, IL-35, TNF-α, TGF-β, and GM-CSF, which indicate their role in immune and hematopoietic modulation (125–127). Over 400 genes are deregulated in MM PCs compared to normal PCs, which could reflect their ability to alter the niche to favor myeloma progression (128).

Macrophages, in the context of the malignant niche, are divided into two groups: classically activated M1 macrophages, and alternatively activated M2 macrophages. The function and phenotype of macrophages depends on their microenvironment, and the general term “tumor associated macrophages” (TAMs) is used to distinguish these cells from normal macrophages (though M2 cells are sometimes classified as TAMs) (129). M1 cells are pro-inflammatory, anti-tumor macrophages activated by bacterial lipopolysaccharides or cytokines produced by Th-1 lymphocytes and secrete IL−1β, IL-6, IL-12, IL-23, TNF-α, ROS, and RNS. M2 macrophages are activated in response to factors produced by Th2 lymphocytes, such as IL-4, IL-10, IL-14, and glucocorticoids. These cells are considered tumorigenic, immunosuppressive and, among others, they produce IL-10, TGF-β, VEGF, matrix metalloproteinases (MMPs), and ARG-1 (109). A high frequency of TAMs was associated with worse prognosis and treatment resistance in MM (130). It was also proved that TAMs cooperate with other cells in the niche. They can mimic ECs in MM and promote neovascularization through VEGF and FGF−2 stimulation (131). Macrophage chemotaxis towards MM BM niche and shift to the tumorigenic M2 phenotype is mediated *via* CCL2, CCL3, CCL14, CXCL12, CSF-1, GM-CSF, MSP, PDGF, and TGF−β,which are produced by MM-associated MSCs (109).

MDSCs were confirmed as immune system inhibitors in cancer patients. These cells express typical CD33+ CD11b+ HLA-DR-/low immunophenotype, with further subdivision into CD15+ granulocytic (G-MDSCs) or CD14+ monocytic (M-MDSCs) subsets (132). It was proposed that G-MDSCs differentiate into tumor-associated neutrophils (TANs) and, similarly, that M-MDSCs are precursors of TAMs. However, possible polarization of normal neutrophils into TANs in the TME is also discussed (133). Increased frequency of MDSCs was found in MM patients, which was also correlated with disease progression and therapy outcome (134, 135). MDSCs produce ROS, RNS, and ARG1, which in detail impair the function of the CD3 T-cell co-receptor participating in the activation of both CD4+ and CD8+ T-cells. Also, MDSCs downregulate the expression of L-selectin (CD62L), thus decreasing T−cell trafficking to the malignant niche (133, 136, 137). Overall, an inhibitory role of MDSCs on the function of NK cells was proved by the co-culture of NK cells with MDSCs, which resulted in the downregulation of activating receptors, decreased secretion of IFN−γ, and decreased degranulation (138). Furthermore, data suggesting a pro-angiogenic potential of MDSCs in MM were published (139). RNS and membrane-bound TGF-β are among the MDSC-derived factors inhibiting NK cell function (140, 141). Overall cooperation of cells present in the malignant niche is reflected by a confirmed ability of MM MSCs to induce the upregulation of TNFα, ARG1, and pro-angiogenic PROK2 in MDSCs (135).

CD3+CD4+CD25+ Tregs are important modulators of normal immune response. The role of Tregs in MM progression seems to be a matter of discussion due to contradictory data. Both decreased and increased Treg frequency can be detected in MM. Increased Tregs were associated with the disease progression, but contradictory data are published too. Nevertheless, these cells clearly contribute to dysfunctional immunity in MM, though the role seems to be heterogeneous (142–146). IL-10 and TGFβ are probably the most discussed cytokines produced by Tregs that may contribute to pathological features of MM BM (144). One of the key Treg-related aspects to maintain functional immunity in MM and tumors in general is a balance in the Treg vs. CD4+ T-helper 17 (Th17) cell ratio. Th17 cells contribute to the development and progression of chronic immune diseases, and cancer, by overall immune regulation and production of IFN-γ, TNF-α, IL-10, IL-17, IL-21, IL-22, and IL-26. It seems that the Treg/Th17 differentiation axis is skewed in MM by elevated levels of IL-6 and TGFβ. In the presence of TGFβ alone, naive T cells that express Foxp3 and differentiate into Tregs Th17 cells are generated in the combination of TGF-β and IL-6, or IL-21 (146). Again, contradictory data have been published on the topic of the Treg/Th17 cell relationship to MM prognosis, and further clarification is needed. Nonetheless, Th17 cells produce high levels of IL-17, which was proved to promote growth of MM cells *in vitro* and *in vivo (* 147, 148).

Without a doubt, MM niche is a deeply complex environment contributing to disease progression and persistence through modulation of the immune response. Nevertheless, only limited data are published about how individual components affect the function of NK cells, which will be discussed in the next chapter.

## NK Cells in the Myeloma Niche

**Figure 4:**
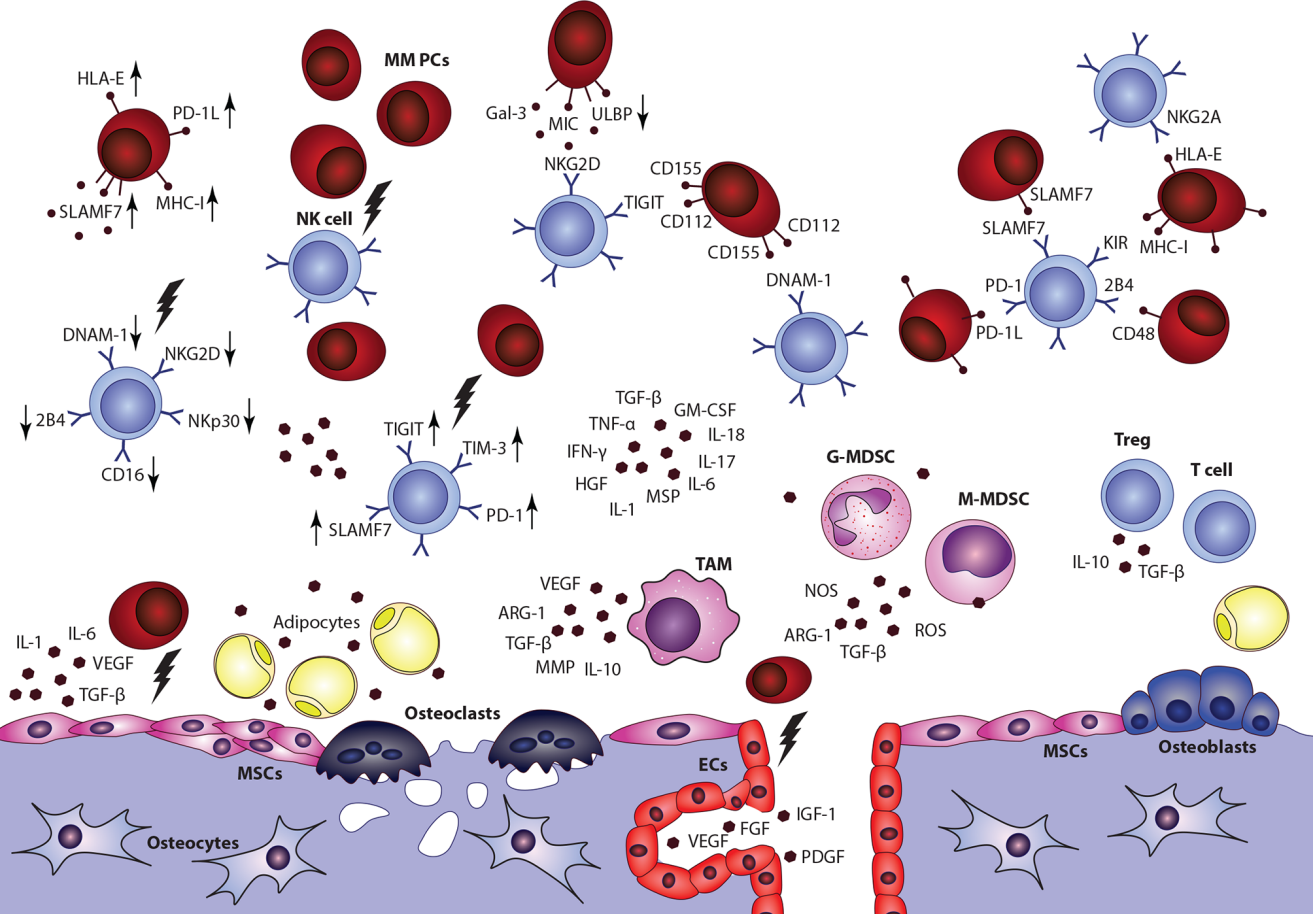
Impact of multiple myeloma bone marrow microenvironment on the overall NK cell function is a complex topic, with only limited data available. Overall decrease of NK cells frequency, accumulation of CD56bright CD16-cytokine producing subset, impaired overall fuctional properties, and alterations of surface effector receptors were connected with the disease and its progression. Nonetheless, detailed data describing interactions between NK cells and individual components in the niche are incomplete. MSCs, mesenchymal stromal cells; ECs, endothelial cells; G-MDSC, granulocytic myeloid-derived suppressor cell; M-MDSC, monocytic myeloid-derived suppressor cell; TAM, tumor associated macrophage.

NK cells act as important regulators in the development and progression of hematological malignancies and their suppressor activity particularly against MM cells was confirmed in many studies (149–152). Nonetheless, significant changes in the distribution of NK subsets and dysfunctions of NK cells were described in MM patients (153, 154). The functional activity of NK cells was also correlated with disease staging (155). Recent studies provided an insight into mechanisms involved in the NK cell−mediated killing of malignant PCs and highlighted the role of interactions between surface effector receptors on the surface of NK cells and specific ligands (156, 157). The recognition of MM cells with activating receptors, including NKG2D, NKp46, and DNAM-1, has been proved (158). Also, a low expression of HLA-1 molecules on malignant PCs and the role of NK inhibitory receptor suppression was demonstrated in MM (150). Downregulation or upregulation of these surface molecules was associated with severe dysfunctions of NK cells in MM. However, details about involved mechanisms between NK cells and individual TME components remain poorly described (**Figure 4**) (159, 160) Data describing the NK cell distribution or functional capabilities in EMD lesions are missing completely, even though NK cell infiltration was connected with better overall survival in solid tumors (161).

### Impairment of NK Cell Development

Since malignant populations are considered to be competitive to non-malignant cells, bone marrow brings a unique insight into the effect of myeloma on the development of healthy immune cells (100). In hematological malignancies, cancer niche disrupts normal hematopoiesis and results in a favorable environment for clonal cells (162). Several publications describe that overall lower percentage of circulating NK cells can be detected in the peripheral blood of MM patients in advanced disease stages with poor prognosis compared to controls, MGUS, and MM with good prognosis (163, 164). However, Pazina et al. recently published that frequencies of NK cells in PB of ND MM and smoldering multiple myeloma (SMM) patients are not significantly decreased compared to healthy donors (HD). Furthermore, overall numbers of PB NK cells in RR MM and post-stem cell transplant (post-SCT) patients were increased in this study, with CD56bright CD16- CD57- stage 4 subset prevailing. This was argued as a possible effect of NK lineage reconstitution after the disease and therapy depletion; hence, it might not reflect the actual disease impact. Frequencies of total NK cells in BM reflected the frequencies in PB, except post-SCT where the frequency was significantly lower in BM. Also, numbers of CD56dim CD57+ cells (representing the terminal and highly active stage 6) are lower in BM compared to PB of ND, RR, and post-SCT MM patients (165). To point out the importance of the terminal stage NK cells, MM patients with higher absolute numbers of CD57+ NK cells were associated with better prognosis compared to patients with higher numbers of more immature CD56bright CD16- CD57- cells (159). Similar to what was published by Pazina et al., overall NK cell numbers and cytotoxic abilities are reduced in B/T-ALL patients as a result of CD56bright CD16- cytokine-producing stage 4 accumulation. In this study, high numbers of cytokine CD56bright CD16- cells were also associated with poor prognosis (166). The accumulation of CD56bright CD16- subset and lower total frequencies of NK cells in the BM reflect that NK cell lineage differentiation is impaired during the progression of hematological malignancies. Nevertheless, scarce information is available in the context of altered NK cell maturation in the environment of malignant BM in general. No study so far has provided detailed data about NK progenitor subset distribution in leukemic or myeloma marrow (167, 168).

In general, early CD34+ CD38- HSCs are not depleted in leukemic marrow since they enter a self-protective quiescence. Nonetheless, leukemic niche affects hematopoietic differentiation leading to reduced levels of CD34+ CD38+ progenitors and subsequent cytopenias (162, 169). There is also evidence that NK maturation in BM is blocked in solid tumors, even though no direct contact is needed between tumor cells and NK cells. One of the reasons is most likely a remotely orchestrated IL-15R downregulation in cancer-altered BM stroma (170). The IL-15/IL-15R axis is indeed an important NK cell development regulator and proliferation inducer, acting *via* IL-2Rβ (CD122), and JAK/STAT, Ras/MEK/MAPK, or PI3K/AKT pathways (171, 172). Mutations in *GATA2* (absent CD56bright cells), *MCM4* (absent CD56dim cells), *IL2-R, JAK3, STAT5*, and *IL-15R* were associated with impaired NK maturation (44, 173, 174).

One of the possible suppressors of IL-15 signaling is prostaglandin E2 (PGE2), which downregulates the γ-chain of the IL-15R complex and subsequently inhibits NK cell function (175). Another candidate is ADAM17, which is activated through the IL-15 axis and reduces NK cell proliferation. Blockade of this metalloproteinase results in increased levels of L-selectin (CD62L) on NK cells, thus supporting the homing of these cells (176). However, not only downregulation of the IL-15R function, but also chronic exposure to IL-15 leads to the NK cell exhaustion (177). Without a doubt, impaired IL−15R/IL−2R signaling and distorted NK cell maturation contributes to the disease progression. Levels of soluble IL-2R in serum and surface expression of IL-2R on malignant PCs or mononuclear cells are significantly increased in MM, which also correlates with the active state of the disease (178, 179). Quite unexpectedly, defects related to the IL-21 axis affect NK cell lytic abilities but not the maturation, even though IL-21 promotes NK cell differentiation (180–182). The maturation capacity of NK cells, together with their ability to respond to the presence of malignant cells, is also reduced with age as the BM stroma deteriorates with time, thus the age of the patients may play a crucial role in this context (183).

The inhibitory role of Tregs in the NK cell differentiation was also confirmed both *in vitro* and *in vivo*. Presence of activated Tregs in the culture of HSCs, which were expanded with NK cell lineage differentiation protocol, led to 90% reduction in NK numbers compared to the control. Similar inhibitory role of Tregs was observed also in mice (184). This phenomenon seems to be caused by increased levels of membrane-bound TGF-β and active TGF-β signaling (184, 185).

Finally, the low numbers of NK cells represent a major issue not only from the view of disease control and progression, but it should also be noted that cancer patients are more susceptible to infections, which are one of the main causes of mortality in MM (186, 187). Understanding the maturation distortion and recovering the generation of mature NK subsets could be crucial for the therapy outcome and patient survival improvement.

### Impairment of NK Cell Localization and Chemotaxis

TME-related downregulation or upregulation of chemotactic factors or their receptors is deeply beneficial for tumor growth, either to attract inflammatory or tumorigenic cells like MDSCs and TAMs, or to repel immunosuppressive cells. As already mentioned, it was well described that tumor infiltration by NK cells contributes to better prognosis. Thus, in hematological malignancies, it is only logical to expect the impairment of NK cell BM localization with consequent efflux to PB (161, 188). In general, several chemotactic receptors are expressed by NK cells, including CCR1, CCR2, CCR5, CCR7, CXCR1, CXCR3, CXCR4, CXCR6, CX3CR1, S1P5, CCRL2, and ChemR23. Indeed, aberrancies in these receptors were connected to lower NK cells’ recruitment to the tumor (189).

**Figure 5:**
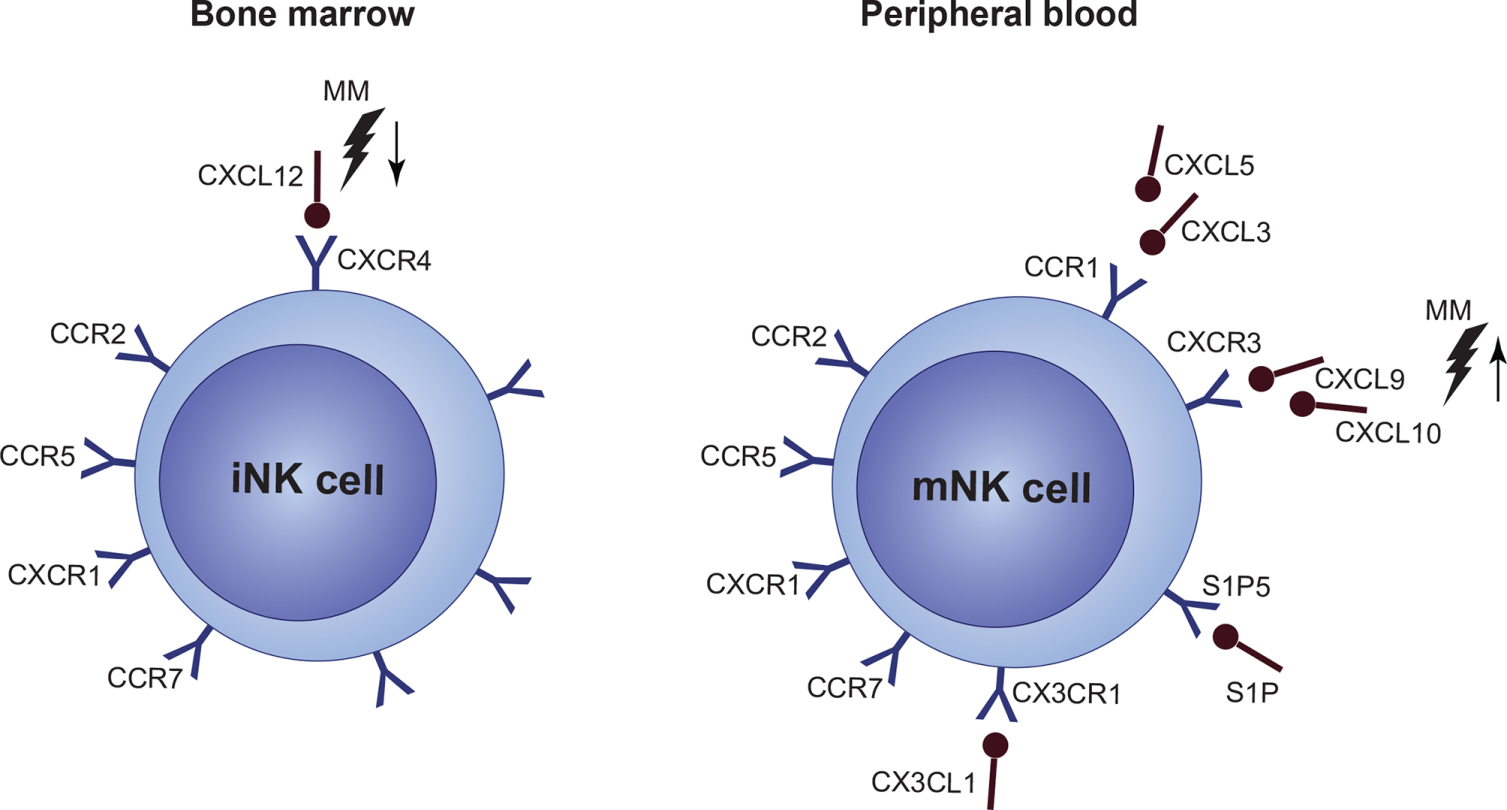
Under normal conditions, immature NK cell (iNK cell) retention in the bone marrow (BM) is mediated via a high expression of CXCR4/CXCL12. Mature NK cells (mNK cell) express only low levels of CXCR4 and high levels of CCR1, CXCR3, CX3CL1 and S1P5 which mediate migration to the periphery. However, expression of individual chemokine receptors, or their ligands, as well as overall function of a crucial CXCR4/CXCR3 signaling, is impaired in multiple myeloma (MM). Occurring CXCL12 upregulation, together with CXCL9 and CXCL10 upregulation during the disease progression suggest, that NK cells are forced out of the BM by factors present in the MM niche. Furthermore, CXCR4/CXCR3 axes seems to be also crucial for the NK cell lineage development.

CXCR4 is one of the key regulators of NK cell BM localization, and it is expressed in high levels by NK progenitors. With decreasing CXCR4 expression in mature stages, levels of CXCR3, CCR1, and CX3CR1 increase, whereas reduced CXCR4 expression, together with S1P5 activation, is necessary for NK cells to exit to the periphery and vice versa (190–192). In general, dysregulation in the CXCR3 and CXCR4 axes was connected to defective BM localization of NK cells in BM. Both of these pathways are closely connected. CXCR3 triggering possibly counteracts CXCR4-mediated BM retention by limiting the CXCR4 responsiveness. Only limited data are available about NK cell disrupted chemotaxis, BM localization and retention in MM, or in other hematological malignancies. In MM BM, several chemokine ligands engaging in NK cell BM localization show a disbalance, including increased levels of CXCL9 and CXCL10 (CXCR3 ligands) and decreased levels of CXCL12 (CXCR4 ligand) (**Figure 5**). Levels of CCL3, CCL5, and CX3CL1 ligands are most likely not subjected to any changes (193).

In other cancers, CXCL12 was confirmed to be downregulated, together with CXCR2 reduction on the surface of NK cells, though data suggest that these changes occur on the post-translational level (194). Another study revealed that tumor tissues tend to overexpress CXCL3 and CXCL5, while expression of CXCL1, CXCL2, and CXCL7 decreases (195). There are also hints, that deregulated CXCR3 signaling in malignant PCs could play a role in MM to EMD progression, although this needs to be confirmed (196). Also, IFN-γ-mediated CXCR3 activation was associated with lower overall survival, and it was proposed as an independent prognostic factor in MM (197). Indeed, inhibition of the CXCR3 axis resulted in better efficacy of IL-15 activated NK cells against malignant PCs (198). CXCR4 was proved to be downregulated in metastatic cells, which also demonstrates its role in malignancy dissemination (199).

Besides, it seems that obstructions in NK cell chemokine signaling and BM/PB localization are connected to the altered NK cell development and the prognosis-related CD56bright CD16- subset accumulation (as discussed previously). About 10%–20% of BM NK cells are localized in proximity to CXCL12 producing osteoblasts and reticular cells that are also able to express IL-15 and IL-15R. This localization is also dependent on the integrin chain α4 (200). Moreover, it was proved that the CXCR4/CXCL12 axis is essential for NK cell development in mice (201).

Chemokine signaling also actively participates in the recruitment of immune suppressor cells. CCR2 and CCR5 contribute to the migration of TAMs and MDSCs into the TME, whereas Tregs with higher expression of CXCR4 are attracted to the TME by their ligands CCL17 and CCL22, which can be produced by TAMs and cancer cells themselves (202). Further research is necessary to understand the chemokine ligand/receptor interactions between NK cells and TME. For example, studies evaluating chemokine/ligand expression profiles on MM cells and NK cell subsets in both BM and PB would probably uncover striking details regarding the role of chemokines in NK cell development and functional impairment, as well as MM-to-EMD progression.

### NK Cell Inhibitory Receptors in MM

Blockade of the checkpoint axis PD-1/PD-1 ligand (PD-1/PD-1L) involved in the inhibition of the immune response has been discussed lately, although this therapy alone seems to be ineffective in MM and combination with other treatment approaches is necessary (203–205). In cancer, expression of PD−1L1 by tumor cells is considered an evasion mechanism promoting the suppression of immune cells (206). Malignant PCs in MM were shown to express higher levels of PD-1L compared to HD or MGUS patients, and significant upregulation can be observed in RR MM patients. Also, PD-1L expression on malignant PCs was connected with resistance to anti−myeloma agents, and the expression of this ligand on PCs was proposed as a marker of poor prognosis in combination with other factors such as age and cytogenetics (205, 207, 208).

Expression of PD-1 was confirmed on the surface of NK cells in MM while undetectable on healthy NK cells (209, 210). Indeed, it was proved that PD-1/PD-1L negatively regulates NK function. However, in this study, at least low levels of PD-1 were also detected on normal circulating or resting NK cells (211). To highlight the therapeutic potential, one study showed that inhibition of PD1/PD-1L signaling in NK cells can increase the degranulation or cytokine-producing ability *in vitro (* 212). Of note, the data reflect the importance of a cautious approach during the flow cytometric detection of PD-1 and subsequent data evaluation since studies reported variable (low or none) levels of PD-1 on healthy NK cells. Furthermore, Pazina et al. encountered difficulties in the detection of any levels of PD-1 even in myeloma samples (165). A recent study proved that PD-1 mRNA and cytoplasmic PD-1 protein can be detected in NK cells, which suggests that surface PD-1 expression is inducible; hence, flow cytometry may provide variable data (213). There are data indicating the ominous role of the TME cellular compartment in PD-1/PD-L axis-related impairment of NK cells. PD-1L-positive MDSCs are present in higher frequency in cancer patients (214, 215). Furthermore, PD-1L expression in MM cells can be also induced by BM MSCs-derived IL-6, with subsequent engagement of JAK/TAT and MEK signaling (208, 216). Use of the JAK inhibitors (ruxolitinib) in MM truly downregulates PD-1L expression in malignant PCs and makes them more susceptible to lenalidomide and steroids (217). Quite curiously, TGF-β, which is abundantly present in the MM niche, seems to have no effect on the expression of PD1-L1 and PD-L2 (218). However, other factors like IL-2, IL-7, and IL-15 were proved to upregulate levels of PD-1L (219). IL-2, IL-7, IL-15, IL-18, and IL-21 are able to upregulate the expression of surface PD−1 too, while IFN-α promotes the transcription of PD-1 (220, 221). HIF-1α is also directly involved in the PD-1L upregulation, which, together with the inhibitory role of HIF-1α in NK cells, propose a multilevel role of hypoxia in MM progression (222, 223).

CD94/NKG2A is an HLA-E-binding molecule recognized as an immune checkpoint like PD-1. Expression of this inhibitory receptor is increased in cancer-associated NK cells, which contributes to their exhaustion (224). Interestingly, HLA-E overexpression in tumors was connected to both poor and good prognosis (225, 226) Although there are data suggesting that the expression of NKG2A is not detrimental for the anti-MM activity of *in vitro* activated NK cells, NKG2A is still a valid target for consideration in immunotherapy. High levels of HLA-E in high-risk MM were proposed as a potential therapeutic candidate, and the experimental blocking of NKG2A by antibodies resulted in restored antitumor activity of NK cells (227–230). Among the MM niche factors, IFN-γ was proved to be involved in cancer-related HLA-E overexpression (231). Furthermore, HLA-E may serve to protect TAMs from CD94/NKG2A-mediated cell lysis (232). From the receptor point of view, TGF-β and IL-10 are among factors inducing the expression of NKG2A (224, 233). Also, IL-2 and IL-15 were shown to upregulate the expression of NKG2A, as well as NCRs, NKG2D, DNAM-1, and KIR2DL4 (234).

KIRs are crucial inhibitory regulators of NK cell response acting through interactions with MHC-I molecules, as already described. Tumor cells can temporarily upregulate their surface MHC-I expression to evade NK cell lysis. On the other hand, MHC-I downregulation is also a common mechanism to avoid immune response (235, 236). Masking the pathological origin (i.e., hiding the “missing-self” signal) by upregulating MHC-I levels is also a feature of MM PCs (237, 238). Moreover, increased levels of KIR molecules KIR2DL1 and KIR2DL2 were described on the PB NK cells of MM patients; however, no further details are available about involved mechanisms (61). Two strategies were proposed to exploit the KIR signaling for the therapy (239). The first is represented by HLA/KIR ligand-mismatched transplantation that showed promising results. Healthy donor NK cells provided a better response than NK cells of the patient, which are corrupted by the tumor inhibitory niche (240, 241). The second option is to directly block the KIR receptor with an antibody to inhibit its interaction with HLA ligand. However, in the MM clinical trial of the anti-KIR antibody IPH2101, this approach was ineffective due to the monocytic trogocytosis of KIR molecules, eventually leading to the lack of education and hyporesponsiveness of NK cells (242). In addition, KIR downregulation leading to enhanced NK cell killing can be achieved also by the stimulation with IL-12, IL-15, and IL-18, while the expression can be restored back after 3 days of culture with IL-2, suggesting an interesting possibility of KIR exploitation with cytokine stimulation (243).

Recently, the upregulation of other possible novel therapeutic target molecules TIGIT, TIM-3, ICOS, and GITR on NK cells was proved in both the PB and BM of MM patients, which probably reflects on the additional immune evasion mechanism (165, 244). TIGIT is a newly identified NK cell immune checkpoint binding to PVR (CD155) and nectin-2 (CD112), which are shared ligands with activating molecule DNAM-1 (245). Nectin-2 was found to be overexpressed on MM PCs, and both PVR and nectin-2 expression were associated with poor prognosis in cancer (246, 247). TIGIT inhibition was proved to restore T-cell response in MM (248). Moreover, TIGIT ligands are highly expressed on cells residing in the BM, which also proposes a role of TIGIT signaling in the MM niche-mediated suppression of NK cell function (246). This is supported by the study showing that BM MSCs upregulate PVR on the surface of MM cells by IL-8 secretion (249). Also, a specific role of MDSCs in the TIGIT/CD155 axis was found. Co-culture of MDSCs with NK cells inhibited their cytotoxic abilities; however, this effect can be reversed either by the inhibition of ROS production (which led to upregulation of PVR in MDSCs) or by the blockade of TIGIT (250). Upregulation of TIM-3, a molecule associated with both inhibitory and activating functions, was also linked with cancer progression as well as CD8+ T cell exhaustion (251–254). In NK cells, it was proved that interactions of TIM-3 with its ligands HMGB1, CEACAM, phosphatidylserine, and galectin-9 inhibit the cytokine production and killing abilities (255). CEACAM ligand overexpression was described in MM, and the expression of HMGB1 was connected to therapy resistance and poor prognosis (256, 257). However, CEACAM downregulation was also correlated with cancer progression (258). These findings may only reflect a heterogeneous role of TIM-3 in the regulation of NK cells in cancer. Further investigation of inhibitory receptors is definitely necessary not only in the context of MM.

### NK Cell Activating Receptors in MM

Both increased and decreased expressions of activating receptors were described in MM. These complex phenotypic changes are attributed to chronic ligand exposure and subsequent NK cell exhaustion (165, 259, 260). Ligands of these receptors were confirmed to be upregulated by MM PCs (238).

Downregulation of NKG2D, as well as 2B4/CD244 and NKp30, can be detected on BM NK cells but not in the PB of MGUS/MM patients (261). However, reduced levels of NKG2D (together with DNAM-1 and CD16) can be observed in both PB and BM of RR and post-SCT MM patients (165). These results reflect the fact that NK cell functional alteration is initiated in the MM BM and later, as the disease progresses, functional impairment is reflected even in circulating NK cells. NKG2D activation is induced by MHC-I-related ligands, which are upregulated as a signal of stress or malignant transformation (262). MIC-A, MIC-B, ULBP-1, ULBP-2, and ULBP-3 are well-known ligands for NKG2D. However, cancer cells are able to downregulate and shed these molecules from their surface. It was shown that high volumes of soluble NKG2D ligands, together with exosomes, are released from tumor cells to chronically exhaust T and NK cells (263–265). However, contradictory data were also published showing that another soluble NKG2D ligand, MULT-1, promotes NK cell function and tumor killing in mice. Nonetheless, the question is whether long-term exposure would not lead to effector cell exhaustion as well (266). One of the mechanisms behind the downregulation and shedding of NKG2D ligands is most likely TGF-β-induced expression of MMP2 (218). MIF was also proved to contribute to the transcriptional downregulation of NGD2D in NK cells (267). Moreover, the expression of NKG2D and NKG2D ligands is downregulated by IDO (indoleamine-2,3-dioxygenase) (268). Recently, CAR-NK cells transduced to express NKG2D-CAR showed a very good anti−myeloma efficacy *in vivo*, with minimal activity against healthy cells. Considering the greater efficacy and lesser toxicity compared to CAR-T cells, these are promising results reflecting the possible use of autologous-engineered CAR-NK cells in the treatment of MM (269).

SLAMF7 (CS1, CRACC, and CD319) is a surface signaling lymphocytic activation molecule (SLAM family) expressed on NK cells and PCs (both normal and malignant), while undetectable in other cells, which makes it a valid target for MM therapy (270, 271). Increased levels of surface SLAMF7 on NK cells were correlated with a worse prognosis in MM (165). Moreover, malignant PCs were proved to cleave SLAMF7 from their surface, leading to increased levels of soluble SLAMF7, which can be detected in MM patients, but not in MGUS. Thus, levels of SLAMF7 can be associated with disease progression. Data also confirmed that soluble SLAMF7 promotes MM cell growth *via* interaction with surface SLAMF7 on MM cells, with subsequent activation of ERK and SHP-2 signaling (272). Furthermore, it was predicted that soluble SLAMF7 could potentially interfere with the novel targeted therapy (273). Anti-SLAMF7 elotuzumab was FDA-approved in 2015, and since then, it has shown promising results in clinical studies. Elotuzumab in combination with lenalidomide and dexamethasone or bortezomib showed increased effectiveness and sustained benefit in progression free survival (274–276). In NK cells, elotuzumab binds to the CD16, which mediates ADCC against the anti-SLAMF7 antibody coupled with SLAMF7 on MM cells. Also, other mechanisms of action include NK cell co-stimulation through NKp30 and NKG2D, stimulation of IFN-γ and granzyme B secretion, as well as macrophage−mediated antibody dependent cell phagocytosis (276, 277). Very intriguing are findings indicating that anti-SLAMF7 antibodies disrupt adhesion of MM PCs to MSCs in the BM. This indicates that elotuzumab might be one of the pioneering agents with a multiple-hit strategy, both against malignant PCs as well as MM niche components (267).

As already mentioned, other activating receptors were proved to be downregulated in MM, including DNAM-1, 2B4 (CD244), and CD16 (165, 261). TGF-β was confirmed as one of the factors causing the downregulation of 2B4 and 2B4 adaptor proteins (DAP10 and SAP) (278, 279). Early studies on 2B4 showed an activating function of this receptor leading to increased killing and IFN-γ production; nonetheless, further research also proved an inhibitory role of this molecule (280, 281). Upregulation of 2B4 and downregulation of the associated adaptor protein SAP were related with inhibitory signaling, while downregulation of 2B4 and normal levels of SAP were associated with activating signaling (282). Among others, downregulation of 2B4 results in defective interactions with its ligand CD48. This also affects the co−stimulation of NCRs mediated through the 2B4-CD48 signaling (259). Lately, 2B4, which is also a member of SLAM family, was proposed as a target for immunotherapy, which could potentially have a double-hit impact affecting MM niche similar to SLAMF7. In particular, 2B4 expression was confirmed on MDSCs (283). Downregulation of CD16 logically results in impaired ADCC (259). However, counterintuitively, it was published that shedding of CD16 from the surface of NK cells leads to the positive stimulation of the immune response by engagement of other immune cells (284). Indeed, levels of soluble CD16 in the serum are significantly decreased in patients with MM compared to MGUS or healthy donors. This was also correlated with the disease staging (285).

The NCR family consists of 3 receptors: NKp30 (NCR3), NKp44 (NCR2), and NKp46 (NCR1). These molecules were originally categorized as activating receptors; nonetheless, it seems that different isoforms of NCRs may exist based on the environment, which then deliver either activating or inhibitory response. Blocking of individual NCRs with mAbs is rather ineffective, while effective inhibition caused by a combination of mAbs against multiple NCR receptors suggest a cooperative mechanism in the process of NK cell activation (286, 287). A positive role of NCRs in cancer control was proved by several studies. NKp46 was connected to metastatic prevention and the potentiation of NK cell antitumor activity by increased IFN-γ production (288, 289). Nevertheless, the activity and function of NCRs can be downregulated in cancer, which, particularly in NKp46, is associated with the progression of malignancy too (290, 291). In MM, NKp30 was proved to be downregulated on BM NK cells, but not in the PB (261). Also, increased expression of NKp30 (on CD56dim CD16+ subset) and NKp44 (CD56bright CD16- and CD56dim CD16+) and decreased expression of NKp44 (CD56bright CD16-) can be observed on PB NK cells in RR MM, but it is not possible to detect any similar changes in ND MM. Again, these data support the fact that functional NK cell properties are impaired preferentially in the site of disease manifestation, and that the impairment is minor (or the function is restored) in circulating NK cells during early states of MM. However, as the disease progresses, further dysfunctional evolution is reflected even in PB NK cells (165). NCRs can be activated by different viral and bacterial ligands, growth factors, ECM−derived and membrane-derived components, or stress related ligands (286). In cancer, several molecules were proved to play a role in NCR activation and cancer cell elimination, though data related to NCR ligands in cancer cells is scarce. B7−H6, BAG-6, and Galectin-3 were confirmed as NKp30 ligands. PCNA, NKp44L (a MLL5-variant protein), and PDGF-DD (platelet-derived growth factor isoform) are among the ligands of NKp44 that might play a role in cancer cell elimination (292–294). However, similarly to what was described in NKG2D ligand shedding, cleavage of B7-H6 ligands by the ADAM-10 and ADAM-17 MMP-related mechanism was showed to chronically exhaust NK cell actions mediated through the NKp30 receptor (295, 296). Galectin-3 can also be released in soluble form by cancer cells to inhibit the NKp30 function (297). Furthermore, tumor-derived TGF-β is one of the factors involved in NKp30 downmodulation (298). In hypoxic conditions, NK cells upregulate HIF−1α, and, curiously, maintain the killing abilities mediated *via* CD16. Nonetheless, the function of activating receptors, including NKp30, NKp44, and NKp46, is impaired (299). NCR-related therapeutical options are not clearly elucidated, though NKp30 was proposed as a target for immunotherapy. However, only CAR-T cells targeting this receptor has been explored, and data relevant to anti-NKp30 mAbs are still missing. Further research is needed regarding the NCR impairment, ligand identification and expression, as well as possible therapeutic options (300).

### Impact of Anti-Myeloma Therapy on NK Cells

As already mentioned, NK cells are important cells mediating the anti-tumor effect of novel mAbs used in the treatment of MM, such as daratumumab, isatuximab, or elotuzumab, and induction of ADCC represents one of several important mechanisms of action induced by these antibodies. Moreover, additional effects of daratumumab mediated *via* NK cells were described, including monocyte activation, phagocytosis, and increased T-cell costimulatory abilities. Hence, any disruption of NK cell immune function might be of great concern and the overall impact of anti-myeloma therapy, including the above-mentioned mAbs, immunomodulatory drugs (IMiDs), or proteasome inhibitors, on NK cells needs to be studied thoroughly (301).

Since CD38 is expressed on the surface of NK cells as well, the question of whether anti-CD38 agents negatively affect or even possibly kill NK cells was raised. Indeed, it was described that daratumumab depletes CD38+ MDSCs, B cells, and Tregs (302). Data published 2 years later confirmed that CD38+ NK cells are also subjected to ADCC induced by daratumumab bound on their surface, which suggested an alarming issue of anti-CD38 therapy (303). A significant reduction of NK cell numbers can be detected in PB and BM of MM patients after initiation of the daratumumab-containing therapy, with the persistence of low NK cell counts during the whole course of the treatment. Nonetheless, no adverse effects on the overall efficacy of the therapy or function of NK cells were discovered. Furthermore, additional immunomodulatory mechanisms of daratumumab participating in the overall therapy efficacy were shown, including increased frequency of CD8+ T cells with preferential generation of effector memory subset (304, 305). Isatuximab was described to mediate even stronger efficacy in the killing of target cells compared to daratumumab, and also the drug was confirmed to induce apoptosis of Tregs with higher CD38 expression than other T cells (28). Similar to daratumumab, reduction of NK cells can be observed after isatuximab application as well, together with the depletion of CD38high B-lymphoid progenitors. Isatuximab-treated NK cells exhibit deregulation of 70 genes, mostly connected to chemotaxis, cytolysis, and immune defense response (28, 95, 306). Anti-SLAMF7 mAb elotuzumab also strongly stimulates NK cell activation, induction of ADCC, and degranulation *via* engagement of CD16. Calcium signaling costimulation triggered by engagement of NKp46 and NKG2D in CD16-independent manner is also activated by this antibody. Regimens containing elotuzumab plus lenalidomide or bortezomib showed promising results, while no adverse effects of elotuzumab on the overall function or frequency of NK cells were observed (307).

Furthermore, proteasome inhibitors such as bortezomib or carfilzomib were described to potentiate NK cell cytotoxicity against MM cells, while no considerable adverse effects on NK cells were reported. Sensitization by downregulation of HLA-I molecules on the surface of malignant PCs is one of the involved mechanisms. Other mechanisms were revealed in studies involving other types of cancer, including bortezomib-induced upregulation of NK cell activating receptor ligands (MIC-A/B, ULBP-1) or ligands related to the death receptor signaling (Fas, DR-5) (308–310).

Immunomodulatory drugs (IMiDs), such as thalidomide, lenalidomide, and pomalidomide, significantly improved therapy outcome in the past two decades and represent indispensable agents that are used to treat MM (311). These agents exhibit pleiotropic anti-MM potential, including anti-angiogenic, anti-inflammatory, immunomodulatory, and anti-proliferative effects (312). In theory, earlier it was proposed that IMiDs could enhance impaired function of immune cells. As a matter of fact, studies confirmed that increased numbers of NK cells can be detected in patients receiving thalidomide therapy, and the positive effect of IMiDs on costimulation of T cells, NK cell proliferation, and their cytotoxic abilities was confirmed as well. Upregulation of IL-2 signaling, along with upregulation of PVR and MIC-A ligands, was discovered to participate in the IMiD-mediated stimulation of NK cells (312–314). Nonetheless, no positive effect of lenalidomide on NK cell activation, degranulation or secretion of IFN-γ or MIP1-β was observed in the study that was monitoring NK cell activity and functionality in 10 MM patients treated with lenalidomide-containing regimen and then maintained with lenalidomide. Progressive post-maintenance NK cell lineage normalization was observed, albeit this was possibly caused by the chemotherapy discontinuation (315). On the other hand, a positive effect of pomalidomide on innate lymphoid cells (ILCs), which are recently discussed lymphoid cells with antitumor potential, was described. Results indicate that pomalidomide leads to enhancement of ILC function through the stimulation of IFN-γ production as well as downregulation of Ikzf1 and Ikzf3, which are transcription factors essential for MM cell proliferation. Similar degradation by ubiquitination of Ikzf1 and Ikzf3 was confirmed by lenalidomide (316, 317).

## Discussion

Defects of NK cell cytokine production, chemotaxis, maturation, effector molecule expression, and related target recognition and killing are described in the context of MM BM or TME in general, although clearly there are large gaps in current knowledge. Unfortunately, some data are even contradictory probably due to the complexity and heterogeneity of the malignant niche and occurring interactions. An overall disruption of NK cell function was correlated with MM and cancer progression; thus, potentiating and restoring the proper NK cell abilities, maturation, and BM localization, as well as normalizing the BM niche, are for sure among the goals for future improvement of patients’ survival and quality of life.

Data covering interactions between NK cells and individual cellular or non-cellular components of the MM niche, which would describe particular mechanisms of NK cell functional impairment, are extensively incomplete. Furthermore, additional information about disrupted NK cell chemokine signaling during MM progression are needed. Data describing the distribution of NK progenitors in the malignant marrow in general are missing completely. Similarly, there is only limited information about mechanisms behind the NK cell maturation distortion during disease progression in the BM. Restoring the generation of fully functional NK cells with normal chemotactic abilities may be critical for the future improvement of therapeutic options. Furthermore, data related to NK cell immune monitoring and expression profiles of surface effector molecules in the EMD are missing. These would provide critical information about the functional capacities of these cells, as well as about levels of potential targets.

Altogether, NK cells and their surface effector molecules represent a tempting therapeutic target in MM and other malignancies, although recent data suggest that combination with conventional protocols is needed in the present. Thus, further research that would uncover all the possible interactions between these receptors, their cognate ligands, as well as interfering factors and cells in the malignant niche is necessary. Moreover, all the data highlight the necessity of further research in the field of IMiDs as well as novel mAbs and proteasome inhibitors used for the treatment of MM. Their mechanisms of action or impact on NK cells or ILCs is still not fully understood. Promising results were published, but unfortunately, most of the available data were generated by *in vitro* or *in vivo* assays, and studies involving MM patients are scarce.

## Author Contributions

All authors made a substantial contribution to the manuscript preparation. OV prepared graphical figures, and conceived and wrote the review. JB, BM, TJ, and RH commented and edited first versions of the manuscript and participated on the final version. All authors contributed to the article and approved the submitted version.

## Funding

This work has been supported by the project Cell Coolab Ostrava—Research and Development Center for Cell Therapy in Hematology and Oncology (No. CZ.02.1.01/0.0/0.0/17_049/0008440), European Regional Development Fund—Project ENOCH (No. CZ.02.1.01/0.0/0.0/16_019/0000868, Student’s grant system SGS15/PrF/2021 University of Ostrava and Institutional support University Hospital Ostrava MH CZ - DRO (FNOs/2021).

## Conflict of Interest

The authors declare that the research was conducted in the absence of any commercial or financial relationships that could be construed as a potential conflict of interest.

## Publisher’s Note

All claims expressed in this article are solely those of the authors and do not necessarily represent those of their affiliated organizations, or those of the publisher, the editors and the reviewers. Any product that may be evaluated in this article, or claim that may be made by its manufacturer, is not guaranteed or endorsed by the publisher.

## Acknowledgments

The authors would like to thank Shira Timilsina Godfrey, M.D., for the language correction.
